# Tetra-μ-acetato-κ^8^ *O*:*O*′-bis­[(3,5-di­methyl-1*H*-pyrazole-κ*N* ^2^)­copper(II)]

**DOI:** 10.1107/S1600536811032600

**Published:** 2011-08-27

**Authors:** Juanita van Wyk, Bernard Omondi, James Darkwa

**Affiliations:** a Department of Chemistry, University of Johannesburg, PO Box 524 Auckland Park, Johannesburg, 2006, South Africa; b School of Chemistry, University of KwaZulu-Natal, Westville Campus, Private Bag X54001, Durban 4000, South Africa

## Abstract

The dinuclear centrosymmetric title compound, [Cu_2_(CH_3_CO_2_)_4_(C_5_H_8_N_2_)_2_], has a distorted square-pyramidal coordination geometry around each Cu^II^ atom in which four O atoms from the bridging acetate ligands form the basal plane while two N atoms from the pyrazole ligands occupy the apical positions. The crystal has two half mol­ecules in the asymmetric unit with a Cu⋯Cu distance of 2.6762 (4) Å. Disorder was found for two O atoms and two C atoms of one acetate ligand and refined with occupancies of 0.265 (7) and 0.735 (7). The crystal also features mol­ecules linked through two N—H⋯O hydrogen bonds resulting in one-dimensional chains extending along the crystallographic *b* axis.

## Related literature

For the properties and applications of 1*H*-pyrazolyl-3,5-substituted ligands, see: Deka *et al.* (2006 ▶); Guzei *et al.* (2003 ▶); Mohlala *et al.* (2005 ▶); Nelana *et al.* (2008 ▶); Ojwach *et al.* (2005 ▶).  
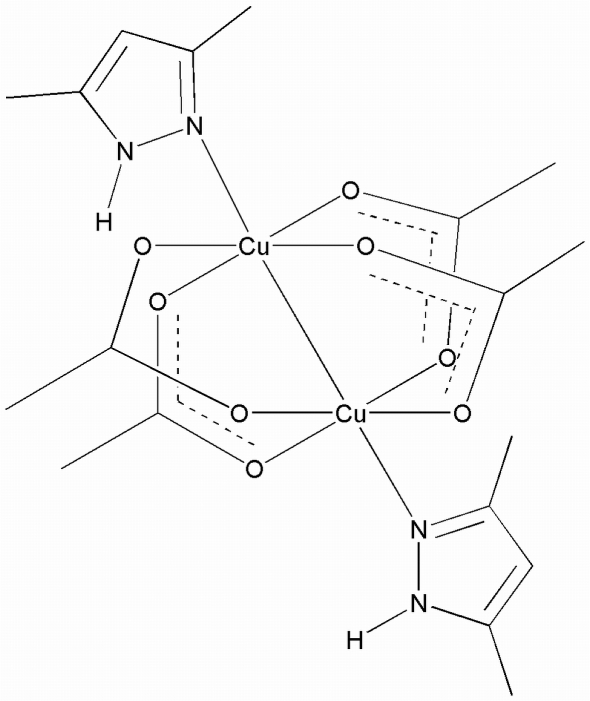


## Experimental

### 

#### Crystal data

[Cu_2_(C_2_H_3_O_2_)_4_(C_5_H_8_N_2_)_2_]
*M* *_r_* = 555.52
Triclinic, 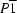

*a* = 8.1125 (4) Å
*b* = 13.6429 (7) Å
*c* = 13.7755 (7) Å
α = 61.571 (1)°
β = 87.449 (1)°
γ = 82.354 (1)°
*V* = 1328.55 (12) Å^3^
*Z* = 2
Mo *K*α radiation
μ = 1.64 mm^−1^
*T* = 100 K
0.39 × 0.16 × 0.10 mm

#### Data collection

Bruker X8 APEXII 4K Kappa CCD diffractometer
Absorption correction: multi-scan (*SADABS*; Bruker, 2007 ▶) *T*_min_ = 0.566, *T*_max_ = 0.853
17190 measured reflections
6578 independent reflections
5934 reflections with *I* > 2σ(*I*)
*R*_int_ = 0.022

#### Refinement

*R*[*F* ^2^ > 2σ(*F* ^2^)] = 0.031
*wR*(*F* ^2^) = 0.089
*S* = 1.06
6578 reflections
310 parameters
17 restraints
H-atom parameters constrained
Δρ_max_ = 1.28 e Å^−3^
Δρ_min_ = −0.59 e Å^−3^

### 

Data collection: *APEX2* (Bruker, 2007 ▶); cell refinement: *SAINT-Plus* (Bruker, 2007 ▶); data reduction: *SAINT-Plus* and *XPREP* (Bruker, 2007 ▶); program(s) used to solve structure: *SHELXS97* (Sheldrick, 2008 ▶); program(s) used to refine structure: *SHELXL97* (Sheldrick, 2008 ▶) and *PLATON* (Spek, 2009 ▶); molecular graphics: *DIAMOND* (Brandenburg & Putz, 2005 ▶), *ORTEP-3* (Farrugia, 1997 ▶); software used to prepare material for publication: *WinGX* (Farrugia, 1999 ▶).

## Supplementary Material

Crystal structure: contains datablock(s) global, I. DOI: 10.1107/S1600536811032600/hg5077sup1.cif

Structure factors: contains datablock(s) I. DOI: 10.1107/S1600536811032600/hg5077Isup2.hkl

Additional supplementary materials:  crystallographic information; 3D view; checkCIF report

## Figures and Tables

**Table 1:** Hydrogen-bond geometry (Å, °)

*D*—H⋯*A*	*D*—H	H⋯*A*	*D*⋯*A*	*D*—H⋯*A*
N2—H2⋯O5	0.88	1.93	2.785 (2)	163
N3—H3⋯O3	0.88	2	2.847 (2)	163

**Fig. 1.**
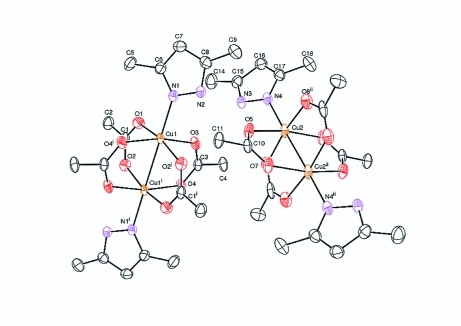
View of (I) (50% probability displacement ellipsoids) with H atoms presented as small spheres of arbitrary radii.

**Fig. 2.**
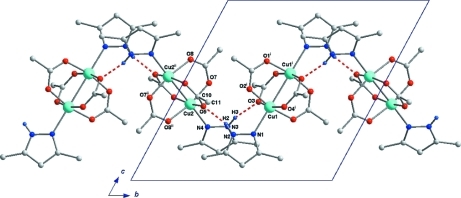
N—H···O hydrogen bond interactions in the crystal structure of (I). [Symmetry operators: (i) = 1 - x, 1 - y, 1 - z and (ii) = 1 - x, -y, 1 - z]

## supplementary crystallographic information

### Comment

Pyrazolyl ligands containing a carbonyl linker has been utilized to prepare a number of coordination compounds with palladium salts (Guzei *et al.*, 2003; Mohlala *et al.*, 2005, Ojwach *et al.*, 2005). In these compounds the pyrazolyl carbonyl moiety appear to be robust enough to avoid hydrolysis. However in a few instances the presence of metal ions like Cu(II) (Deka *et al.*, 2006) and Pd(II) (Nelana *et al.*, 2008) appear to catalyze the hydrolysis of the benzoyl fragments. We have observed similar hydrolysis when reacting copper(II) acetate with (3,5-dimethyl-pyrazol-1-yl)-*o*-benzoyl-methane. The title compound formed from this reaction is the subject of this report. The half "solvent" molecule excluded from the structure had a total number of 30.7 electrons which is approximately half the total number of electrons that acetophenone has.

Compound (I) crystallizes with two half molecules in the assymetric unit. The compound is dinuclear with each of the Cu atoms coordinated to four O atoms and a N atom from the pyrazole ligand. The O atoms are from acetate ions, all in the equatorial positions of a slightly distorted octahedral geometry around the Cu atoms. The N atom is bound *trans* to the Cu—Cu vector completing a the distorted octahedral geometry as axial ligands.

The crystal structure of (I) is composed of two N—H···O hydrogen bonded chains (Table 1) that extend in the crystallographic *b* axis (Fig. 2).

### Experimental

A mixture of copper(II) acetate monohydrate (0.20 g, 1 mmol) and (3,5-dimethy-pyrazol-1-yl)-*o*-benzyl-methane (0.20 g, 1 mmol) was refluxed in methanol (20 ml) for 4 h. The bluish-green mixture turned deep green during the course of the reaction and upon removal of the solvent a green solid residue was obtained. Recrystallization from a methanol:ethylacetate (1:2) mixture produced X-ray quality crystals after several days. Yield = 0.36 g, 59%

### Refinement

The methyl, methine and aromatic H atoms were placed in geometrically idealized positions and constrained to ride on their parent atoms, with C—H = 0.95 Å and *U*_iso_(H) = 1.2*U*_eq_(C) for aromatic, C—H = 0.98 Å and *U*_iso_(H) = 1.2*U*_eq_(C) for CH_3_ and N—H = 0.88 Å and *U*_iso_(H) = 1.2*U*_eq_(C) for NH. Half a molecule of acetophenone that resided on an inversion center was grossly disordered and was was excluded using the SQUEEZE subroutine in *PLATON* (Spek (2009).

### Crystal data

**Table:**

[Cu_2_(C_2_H_3_O_2_)_4_(C_5_H_8_N_2_)_2_]	*Z* = 2
*M**_r_* = 555.52	*F*(000) = 572
Triclinic, *P*1	*D*_x_ = 1.389 Mg m^−^^3^
Hall symbol: -P 1	Mo *K*α radiation, λ = 0.71073 Å
*a* = 8.1125 (4) Å	Cell parameters from 17682 reflections
*b* = 13.6429 (7) Å	θ = 2.5–28.4°
*c* = 13.7755 (7) Å	µ = 1.64 mm^−^^1^
α = 61.571 (1)°	*T* = 100 K
β = 87.449 (1)°	Block, green
γ = 82.354 (1)°	0.39 × 0.16 × 0.1 mm
*V* = 1328.55 (12) Å^3^	

### Data collection

**Table:**

Bruker X8 APEXII 4K Kappa CCD diffractometer	5934 reflections with *I* > 2σ(*I*)
graphite	*R*_int_ = 0.022
φ and ω scans	θ_max_ = 28.4°, θ_min_ = 2.5°
Absorption correction: multi-scan (*SADABS*; Bruker, 2007)	*h* = −10→10
*T*_min_ = 0.566, *T*_max_ = 0.853	*k* = −18→17
17190 measured reflections	*l* = −18→18
6578 independent reflections	

### Refinement

**Table:**

Refinement on *F*^2^	Primary atom site location: structure-invariant direct methods
Least-squares matrix: full	Secondary atom site location: difference Fourier map
*R*[*F*^2^ > 2σ(*F*^2^)] = 0.031	Hydrogen site location: inferred from neighbouring sites
*wR*(*F*^2^) = 0.089	H-atom parameters constrained
*S* = 1.06	*w* = 1/[σ^2^(*F*_o_^2^) + (0.0503*P*)^2^ + 0.7634*P*] where *P* = (*F*_o_^2^ + 2*F*_c_^2^)/3
6578 reflections	(Δ/σ)_max_ = 0.012
310 parameters	Δρ_max_ = 1.28 e Å^−^^3^
17 restraints	Δρ_min_ = −0.59 e Å^−^^3^

### Special details

**Table:**

Experimental. Diorder: Disorder was found for two O atoms and two C atoms of one acetate ligand in which is not an uncommon situation. The disorder was modelled for two O–, two C– and H-atoms using distance restraints and PART instructions and the total occupancy at each atom site was kept as 1 during the refinement. DELU and SIMU constraints and restraints were used on the disordered atoms. All carbon atoms involved in disorder were modelled with anisotropic thermal parameters and refined with occupancies of 0.265 (7) and 0.735 (7). The "solvent" molecule resided in a special position and looked disordered as well. Modelling the disorder only distabillized the refinement. As a result, the moleculed was removed using the SQUEEZE subroutine in *PLATON* giving an *R* factor of 3.0%. H-atom Placement: All H-atoms were placed in idealized locations and refined as riding with appropriate thermal displacement coefficients *U*_iso_(H) = 1.2 or 1.5 times *U*_eq_(bearing atom).
Geometry. All e.s.d.'s (except the e.s.d. in the dihedral angle between two l.s. planes) are estimated using the full covariance matrix. The cell e.s.d.'s are taken into account individually in the estimation of e.s.d.'s in distances, angles and torsion angles; correlations between e.s.d.'s in cell parameters are only used when they are defined by crystal symmetry. An approximate (isotropic) treatment of cell e.s.d.'s is used for estimating e.s.d.'s involving l.s. planes.
Refinement. Refinement of *F*^2^ against ALL reflections. The weighted *R*-factor *wR* and goodness of fit *S* are based on *F*^2^, conventional *R*-factors *R* are based on *F*, with *F* set to zero for negative *F*^2^. The threshold expression of *F*^2^ > σ(*F*^2^) is used only for calculating *R*-factors(gt) *etc*. and is not relevant to the choice of reflections for refinement. *R*-factors based on *F*^2^ are statistically about twice as large as those based on *F*, and *R*- factors based on ALL data will be even larger.The following ALERTS were generated. Each ALERT has the format test-name_ALERT_alert-type_alert-level.PLAT094_ALERT_2_C Ratio of Maximum / Minimum Residual Density ···. 2.18The solvent molecule was excluded. The remaining electron density peaks are very close to other atoms and make no chemical sense. PLAT910_ALERT_3_C Missing # of FCF Reflections Below Th(Min) ···.. 3 PLAT911_ALERT_3_C Missing # FCF Refl Between THmin & STh/*L*= 0.600 33 PLAT912_ALERT_4_C Missing # of FCF Reflections Above STh/*L*= 0.600 39 PLAT913_ALERT_3_C Missing # of Very Strong Reflections in FCF ···. 1 PLAT002_ALERT_2_G Number of Distance or Angle Restraints on AtSite 10 PLAT003_ALERT_2_G Number of *U*_iso_ or *U^ij^* Restrained Atom Sites ···. 4 PLAT154_ALERT_1_G The su's on the Cell Angles are Equal ·········. 0.00100 Deg.These are noted PLAT301_ALERT_3_G Note: Main Residue Disorder ··················. 13 Perc.See disorder explanation above. PLAT380_ALERT_4_G Check Incorrectly? Oriented *X*(sp2)-Methyl Moiety C18 PLAT605_ALERT_4_G Structure Contains Solvent Accessible VOIDS of. 199 A**3 PLAT869_ALERT_4_G ALERTS Related to the use of SQUEEZE Suppressed ! Solvent molecule was excluded as it was grossly disordered. PLAT764_ALERT_4_G Overcomplete CIF Bond List Detected (Rep/Expd). 1.17 Ratio PLAT860_ALERT_3_G Note: Number of Least-Squares Restraints ······. 17 PLAT961_ALERT_5_G Dataset Contains no Negative Intensities ······. ! Noted.

### Fractional atomic coordinates and isotropic or equivalent isotropic displacement parameters (Å^2^)

**Table:**

	*x*	*y*	*z*	*U*_iso_*/*U*_eq_	Occ. (<1)
Cu1	0.53478 (2)	0.494657 (16)	0.406632 (17)	0.01851 (7)	
O1	0.40264 (18)	0.64415 (11)	0.33807 (11)	0.0291 (3)	
O2	0.34793 (16)	0.65464 (11)	0.49368 (11)	0.0270 (3)	
C1	0.3431 (2)	0.69462 (15)	0.39072 (16)	0.0237 (3)	
C2	0.2597 (3)	0.81398 (16)	0.32348 (18)	0.0329 (4)	
H2A	0.1426	0.8186	0.3437	0.049*	
H2B	0.2666	0.8358	0.2447	0.049*	
H2C	0.3159	0.8648	0.3385	0.049*	
O3	0.32633 (15)	0.42403 (12)	0.43652 (12)	0.0264 (3)	
O4	0.26852 (16)	0.43494 (12)	0.59184 (11)	0.0272 (3)	
N1	0.59519 (19)	0.48564 (13)	0.25736 (13)	0.0227 (3)	
N2	0.61058 (18)	0.38656 (13)	0.25468 (13)	0.0225 (3)	
H2	0.602	0.3213	0.3131	0.027*	
C3	0.2366 (2)	0.40998 (14)	0.51864 (16)	0.0226 (3)	
C4	0.0775 (2)	0.35986 (17)	0.52824 (19)	0.0308 (4)	
H4A	0.0222	0.3494	0.5966	0.046*	
H4B	0.1034	0.2871	0.5294	0.046*	
H4C	0.0036	0.4106	0.4649	0.046*	
C5	0.6131 (3)	0.68424 (17)	0.12252 (19)	0.0398 (5)	
H5A	0.5011	0.7136	0.134	0.06*	
H5B	0.6415	0.7267	0.0447	0.06*	
H5C	0.6935	0.692	0.1687	0.06*	
C6	0.6178 (2)	0.56309 (16)	0.15334 (16)	0.0269 (4)	
C7	0.6457 (3)	0.51248 (17)	0.08502 (16)	0.0303 (4)	
H7	0.6644	0.5488	0.0079	0.036*	
C8	0.6403 (2)	0.39916 (17)	0.15302 (16)	0.0272 (4)	
C9	0.6625 (3)	0.30197 (19)	0.12979 (19)	0.0363 (5)	
H9A	0.7677	0.2549	0.1634	0.055*	
H9B	0.6644	0.3298	0.0498	0.055*	
H9C	0.5701	0.2574	0.1609	0.055*	
Cu2	0.40853 (2)	0.091442 (18)	0.42346 (2)	0.02307 (7)	
O7A	0.338 (2)	0.1009 (10)	0.5625 (7)	0.0308 (6)	0.265 (7)
O8A	0.500 (3)	−0.0504 (12)	0.6844 (12)	0.0307 (7)	0.265 (7)
C12A	0.390 (2)	0.0326 (10)	0.6618 (7)	0.0265 (7)	0.265 (7)
C13A	0.3283 (13)	0.0421 (10)	0.7642 (8)	0.0357 (8)	0.265 (7)
H13A	0.4128	0.0705	0.7894	0.054*	0.265 (7)
H13B	0.308	−0.0321	0.8231	0.054*	0.265 (7)
H13C	0.2247	0.094	0.7457	0.054*	0.265 (7)
O7	0.3327 (7)	0.1281 (3)	0.5399 (3)	0.0308 (6)	0.735 (7)
O8	0.4827 (8)	−0.0262 (4)	0.6696 (4)	0.0307 (7)	0.735 (7)
C12	0.3826 (6)	0.0633 (3)	0.6357 (3)	0.0265 (7)	0.735 (7)
C13	0.3140 (4)	0.0927 (3)	0.7245 (3)	0.0357 (8)	0.735 (7)
H13D	0.3479	0.1646	0.7103	0.054*	0.735 (7)
H13E	0.3576	0.0338	0.797	0.054*	0.735 (7)
H13F	0.1923	0.0988	0.7234	0.054*	0.735 (7)
O5	0.61101 (16)	0.16201 (11)	0.41197 (12)	0.0290 (3)	
O6	0.76410 (15)	0.00869 (11)	0.53847 (12)	0.0264 (3)	
N3	0.23162 (18)	0.33734 (13)	0.29863 (14)	0.0241 (3)	
H3	0.2743	0.3505	0.3483	0.029*	
N4	0.26197 (18)	0.23761 (13)	0.29750 (14)	0.0248 (3)	
C10	0.7459 (2)	0.10916 (14)	0.46471 (16)	0.0217 (3)	
C11	0.8953 (2)	0.17237 (16)	0.43519 (18)	0.0283 (4)	
H11A	0.9293	0.1893	0.3602	0.042*	
H11B	0.9871	0.1262	0.4874	0.042*	
H11C	0.8665	0.2427	0.4387	0.042*	
C14	0.0769 (3)	0.52926 (18)	0.2005 (2)	0.0438 (5)	
H14A	−0.0194	0.5283	0.2464	0.066*	
H14B	0.0469	0.5806	0.1228	0.066*	
H14C	0.1688	0.5548	0.2226	0.066*	
C15	0.1293 (2)	0.41377 (17)	0.21551 (17)	0.0295 (4)	
C16	0.0898 (3)	0.36072 (18)	0.15710 (17)	0.0325 (4)	
H16	0.0191	0.3919	0.0935	0.039*	
C17	0.1746 (2)	0.25240 (17)	0.21025 (17)	0.0291 (4)	
C18	0.1723 (3)	0.1581 (2)	0.1831 (2)	0.0442 (5)	
H18A	0.2449	0.0919	0.2356	0.066*	
H18B	0.2121	0.1813	0.108	0.066*	
H18C	0.0585	0.1397	0.1881	0.066*	

### Atomic displacement parameters (Å^2^)

**Table:**

	*U*^11^	*U*^22^	*U*^33^	*U*^12^	*U*^13^	*U*^23^
Cu1	0.01382 (10)	0.01959 (11)	0.02424 (11)	−0.00497 (7)	−0.00013 (7)	−0.01135 (9)
O1	0.0309 (7)	0.0250 (6)	0.0293 (7)	0.0015 (5)	−0.0050 (5)	−0.0119 (6)
O2	0.0234 (6)	0.0237 (6)	0.0323 (7)	0.0002 (5)	0.0000 (5)	−0.0127 (5)
C1	0.0167 (7)	0.0199 (8)	0.0342 (9)	−0.0051 (6)	−0.0024 (7)	−0.0115 (7)
C2	0.0340 (10)	0.0204 (8)	0.0389 (11)	0.0002 (7)	−0.0056 (8)	−0.0102 (8)
O3	0.0175 (6)	0.0336 (7)	0.0382 (7)	−0.0098 (5)	0.0029 (5)	−0.0238 (6)
O4	0.0212 (6)	0.0345 (7)	0.0314 (7)	−0.0143 (5)	0.0032 (5)	−0.0176 (6)
N1	0.0203 (7)	0.0227 (7)	0.0261 (7)	−0.0055 (5)	0.0016 (5)	−0.0118 (6)
N2	0.0194 (7)	0.0231 (7)	0.0270 (7)	−0.0055 (5)	0.0019 (6)	−0.0129 (6)
C3	0.0142 (7)	0.0206 (8)	0.0342 (9)	−0.0043 (6)	−0.0016 (6)	−0.0132 (7)
C4	0.0189 (8)	0.0351 (10)	0.0491 (12)	−0.0132 (7)	0.0053 (8)	−0.0267 (9)
C5	0.0529 (13)	0.0254 (10)	0.0361 (11)	−0.0120 (9)	0.0088 (10)	−0.0095 (8)
C6	0.0270 (9)	0.0265 (9)	0.0261 (9)	−0.0064 (7)	0.0011 (7)	−0.0108 (7)
C7	0.0313 (10)	0.0348 (10)	0.0241 (9)	−0.0050 (8)	0.0017 (7)	−0.0133 (8)
C8	0.0221 (8)	0.0339 (10)	0.0296 (9)	−0.0051 (7)	0.0004 (7)	−0.0178 (8)
C9	0.0376 (11)	0.0404 (11)	0.0410 (11)	−0.0042 (9)	0.0013 (9)	−0.0276 (10)
Cu2	0.01346 (11)	0.02278 (12)	0.03971 (14)	−0.00579 (8)	0.00376 (9)	−0.01966 (10)
O7A	0.0260 (8)	0.0252 (19)	0.0478 (14)	0.0024 (16)	0.0025 (14)	−0.0243 (13)
O8A	0.0267 (17)	0.029 (2)	0.0418 (16)	−0.0021 (15)	0.0072 (12)	−0.0219 (16)
C12A	0.0203 (10)	0.028 (2)	0.0427 (16)	−0.0108 (16)	0.0121 (15)	−0.0251 (15)
C13A	0.0362 (13)	0.040 (2)	0.0327 (19)	0.0104 (15)	0.0002 (13)	−0.0219 (17)
O7	0.0260 (8)	0.0252 (19)	0.0478 (14)	0.0024 (16)	0.0025 (14)	−0.0243 (13)
O8	0.0267 (17)	0.029 (2)	0.0418 (16)	−0.0021 (15)	0.0072 (12)	−0.0219 (16)
C12	0.0203 (10)	0.028 (2)	0.0427 (16)	−0.0108 (16)	0.0121 (15)	−0.0251 (15)
C13	0.0362 (13)	0.040 (2)	0.0327 (19)	0.0104 (15)	0.0002 (13)	−0.0219 (17)
O5	0.0178 (6)	0.0226 (6)	0.0441 (8)	−0.0066 (5)	−0.0034 (5)	−0.0127 (6)
O6	0.0147 (5)	0.0223 (6)	0.0437 (8)	−0.0053 (5)	0.0016 (5)	−0.0162 (6)
N3	0.0181 (7)	0.0247 (7)	0.0330 (8)	−0.0053 (5)	−0.0005 (6)	−0.0157 (6)
N4	0.0186 (7)	0.0278 (8)	0.0349 (8)	−0.0079 (6)	0.0034 (6)	−0.0194 (7)
C10	0.0153 (7)	0.0212 (8)	0.0353 (9)	−0.0058 (6)	0.0042 (6)	−0.0183 (7)
C11	0.0166 (8)	0.0245 (8)	0.0461 (11)	−0.0078 (6)	0.0045 (7)	−0.0175 (8)
C14	0.0451 (13)	0.0270 (10)	0.0538 (14)	0.0009 (9)	−0.0147 (11)	−0.0148 (10)
C15	0.0233 (9)	0.0293 (9)	0.0334 (10)	−0.0073 (7)	0.0005 (7)	−0.0120 (8)
C16	0.0305 (10)	0.0382 (11)	0.0282 (9)	−0.0103 (8)	−0.0002 (8)	−0.0138 (8)
C17	0.0258 (9)	0.0365 (10)	0.0313 (9)	−0.0118 (7)	0.0051 (7)	−0.0197 (8)
C18	0.0488 (13)	0.0505 (14)	0.0502 (13)	−0.0092 (11)	−0.0038 (11)	−0.0363 (12)

### Geometric parameters (Å, °)

**Table:**

Cu1—O2^i^	1.9691 (13)	Cu2—O7A	2.032 (9)
Cu1—O1	1.9685 (13)	Cu2—N4	2.1585 (17)
Cu1—O4^i^	1.9747 (12)	Cu2—Cu2^ii^	2.6755 (5)
Cu1—O3	1.9879 (12)	O7A—C12A	1.283 (8)
Cu1—N1	2.1492 (16)	O8A—C12A	1.265 (9)
Cu1—Cu1^i^	2.6763 (4)	O8A—Cu2^ii^	1.912 (15)
O1—C1	1.261 (2)	C12A—C13A	1.534 (9)
O2—C1	1.254 (2)	C13A—H13A	0.98
O2—Cu1^i^	1.9691 (13)	C13A—H13B	0.98
C1—C2	1.515 (2)	C13A—H13C	0.98
C2—H2A	0.98	O7—C12	1.236 (4)
C2—H2B	0.98	O8—C12	1.263 (4)
C2—H2C	0.98	O8—Cu2^ii^	1.997 (5)
O3—C3	1.266 (2)	C12—C13	1.522 (4)
O4—C3	1.254 (2)	C13—H13D	0.98
O4—Cu1^i^	1.9747 (12)	C13—H13E	0.98
N1—C6	1.338 (2)	C13—H13F	0.98
N1—N2	1.359 (2)	O5—C10	1.268 (2)
N2—C8	1.342 (2)	O6—C10	1.252 (2)
N2—H2	0.88	O6—Cu2^ii^	1.9657 (12)
C3—C4	1.512 (2)	N3—C15	1.344 (2)
C4—H4A	0.98	N3—N4	1.357 (2)
C4—H4B	0.98	N3—H3	0.88
C4—H4C	0.98	N4—C17	1.340 (3)
C5—C6	1.493 (3)	C10—C11	1.509 (2)
C5—H5A	0.98	C11—H11A	0.98
C5—H5B	0.98	C11—H11B	0.98
C5—H5C	0.98	C11—H11C	0.98
C6—C7	1.404 (3)	C14—C15	1.492 (3)
C7—C8	1.380 (3)	C14—H14A	0.98
C7—H7	0.95	C14—H14B	0.98
C8—C9	1.493 (3)	C14—H14C	0.98
C9—H9A	0.98	C15—C16	1.383 (3)
C9—H9B	0.98	C16—C17	1.392 (3)
C9—H9C	0.98	C16—H16	0.95
Cu2—O8A^ii^	1.912 (15)	C17—C18	1.503 (3)
Cu2—O7	1.949 (3)	C18—H18A	0.98
Cu2—O6^ii^	1.9657 (12)	C18—H18B	0.98
Cu2—O5	1.9756 (13)	C18—H18C	0.98
Cu2—O8^ii^	1.997 (5)		
			
O2^i^—Cu1—O1	167.13 (6)	O5—Cu2—O8^ii^	88.6 (2)
O2^i^—Cu1—O4^i^	90.21 (6)	O8A^ii^—Cu2—O7A	167.1 (4)
O1—Cu1—O4^i^	89.34 (6)	O7—Cu2—O7A	9.9 (3)
O2^i^—Cu1—O3	87.95 (6)	O6^ii^—Cu2—O7A	84.8 (5)
O1—Cu1—O3	89.68 (6)	O5—Cu2—O7A	92.1 (5)
O4^i^—Cu1—O3	167.33 (6)	O8^ii^—Cu2—O7A	158.3 (3)
O2^i^—Cu1—N1	95.41 (6)	O8A^ii^—Cu2—N4	90.8 (3)
O1—Cu1—N1	97.43 (6)	O7—Cu2—N4	93.06 (12)
O4^i^—Cu1—N1	95.81 (6)	O6^ii^—Cu2—N4	95.65 (6)
O3—Cu1—N1	96.84 (6)	O5—Cu2—N4	97.26 (6)
O2^i^—Cu1—Cu1^i^	84.06 (4)	O8^ii^—Cu2—N4	99.45 (13)
O1—Cu1—Cu1^i^	83.11 (4)	O7A—Cu2—N4	101.9 (3)
O4^i^—Cu1—Cu1^i^	83.34 (4)	O8A^ii^—Cu2—Cu2^ii^	88.0 (3)
O3—Cu1—Cu1^i^	83.99 (4)	O7—Cu2—Cu2^ii^	88.14 (11)
N1—Cu1—Cu1^i^	179.00 (4)	O6^ii^—Cu2—Cu2^ii^	84.11 (4)
C1—O1—Cu1	124.01 (12)	O5—Cu2—Cu2^ii^	82.99 (4)
C1—O2—Cu1^i^	123.04 (12)	O8^ii^—Cu2—Cu2^ii^	79.36 (12)
O2—C1—O1	125.59 (17)	O7A—Cu2—Cu2^ii^	79.3 (3)
O2—C1—C2	117.43 (17)	N4—Cu2—Cu2^ii^	178.78 (5)
O1—C1—C2	116.98 (17)	C12A—O7A—Cu2	127.1 (9)
C1—C2—H2A	109.5	C12A—O8A—Cu2^ii^	123.2 (10)
C1—C2—H2B	109.5	O8A—C12A—O7A	122.3 (10)
H2A—C2—H2B	109.5	O8A—C12A—C13A	112.9 (9)
C1—C2—H2C	109.5	O7A—C12A—C13A	124.8 (10)
H2A—C2—H2C	109.5	C12A—C13A—H13A	109.5
H2B—C2—H2C	109.5	C12A—C13A—H13B	109.5
C3—O3—Cu1	122.78 (11)	H13A—C13A—H13B	109.5
C3—O4—Cu1^i^	124.51 (12)	C12A—C13A—H13C	109.5
C6—N1—N2	105.07 (15)	H13A—C13A—H13C	109.5
C6—N1—Cu1	133.35 (13)	H13B—C13A—H13C	109.5
N2—N1—Cu1	121.51 (11)	C12—O7—Cu2	118.6 (3)
C8—N2—N1	112.54 (15)	C12—O8—Cu2^ii^	126.0 (3)
C8—N2—H2	123.7	O7—C12—O8	127.8 (4)
N1—N2—H2	123.7	O7—C12—C13	116.7 (3)
O4—C3—O3	125.35 (16)	O8—C12—C13	115.5 (4)
O4—C3—C4	117.62 (17)	C10—O5—Cu2	124.10 (12)
O3—C3—C4	117.03 (16)	C10—O6—Cu2^ii^	123.70 (11)
C3—C4—H4A	109.5	C15—N3—N4	112.53 (16)
C3—C4—H4B	109.5	C15—N3—H3	123.7
H4A—C4—H4B	109.5	N4—N3—H3	123.7
C3—C4—H4C	109.5	C17—N4—N3	104.94 (16)
H4A—C4—H4C	109.5	C17—N4—Cu2	131.64 (13)
H4B—C4—H4C	109.5	N3—N4—Cu2	123.27 (12)
C6—C5—H5A	109.5	O6—C10—O5	124.78 (16)
C6—C5—H5B	109.5	O6—C10—C11	117.99 (15)
H5A—C5—H5B	109.5	O5—C10—C11	117.23 (16)
C6—C5—H5C	109.5	C10—C11—H11A	109.5
H5A—C5—H5C	109.5	C10—C11—H11B	109.5
H5B—C5—H5C	109.5	H11A—C11—H11B	109.5
N1—C6—C7	110.27 (17)	C10—C11—H11C	109.5
N1—C6—C5	121.39 (18)	H11A—C11—H11C	109.5
C7—C6—C5	128.34 (18)	H11B—C11—H11C	109.5
C8—C7—C6	105.85 (17)	C15—C14—H14A	109.5
C8—C7—H7	127.1	C15—C14—H14B	109.5
C6—C7—H7	127.1	H14A—C14—H14B	109.5
N2—C8—C7	106.27 (17)	C15—C14—H14C	109.5
N2—C8—C9	122.23 (18)	H14A—C14—H14C	109.5
C7—C8—C9	131.49 (19)	H14B—C14—H14C	109.5
C8—C9—H9A	109.5	N3—C15—C16	105.90 (18)
C8—C9—H9B	109.5	N3—C15—C14	122.15 (19)
H9A—C9—H9B	109.5	C16—C15—C14	131.9 (2)
C8—C9—H9C	109.5	C15—C16—C17	106.16 (18)
H9A—C9—H9C	109.5	C15—C16—H16	126.9
H9B—C9—H9C	109.5	C17—C16—H16	126.9
O8A^ii^—Cu2—O7	175.4 (6)	N4—C17—C16	110.47 (18)
O8A^ii^—Cu2—O6^ii^	92.2 (7)	N4—C17—C18	120.98 (19)
O7—Cu2—O6^ii^	89.93 (17)	C16—C17—C18	128.53 (19)
O8A^ii^—Cu2—O5	88.0 (7)	C17—C18—H18A	109.5
O7—Cu2—O5	89.00 (18)	C17—C18—H18B	109.5
O6^ii^—Cu2—O5	167.08 (6)	H18A—C18—H18B	109.5
O8A^ii^—Cu2—O8^ii^	8.8 (4)	C17—C18—H18C	109.5
O7—Cu2—O8^ii^	167.47 (13)	H18A—C18—H18C	109.5
O6^ii^—Cu2—O8^ii^	89.6 (2)	H18B—C18—H18C	109.5
			
O2^i^—Cu1—O1—C1	8.2 (3)	Cu2—O7A—C12A—O8A	3(3)
O4^i^—Cu1—O1—C1	−79.85 (15)	Cu2—O7A—C12A—C13A	−178.1 (12)
O3—Cu1—O1—C1	87.51 (15)	O6^ii^—Cu2—O7—C12	82.6 (4)
N1—Cu1—O1—C1	−175.63 (14)	O5—Cu2—O7—C12	−84.5 (4)
Cu1^i^—Cu1—O1—C1	3.53 (14)	O8^ii^—Cu2—O7—C12	−5.3 (15)
Cu1^i^—O2—C1—O1	4.2 (3)	O7A—Cu2—O7—C12	24 (4)
Cu1^i^—O2—C1—C2	−175.42 (12)	N4—Cu2—O7—C12	178.3 (4)
Cu1—O1—C1—O2	−5.7 (3)	Cu2^ii^—Cu2—O7—C12	−1.5 (4)
Cu1—O1—C1—C2	173.89 (12)	Cu2—O7—C12—O8	1.6 (9)
O2^i^—Cu1—O3—C3	85.70 (14)	Cu2—O7—C12—C13	−177.4 (3)
O1—Cu1—O3—C3	−81.65 (14)	Cu2^ii^—O8—C12—O7	−0.5 (10)
O4^i^—Cu1—O3—C3	3.9 (3)	Cu2^ii^—O8—C12—C13	178.5 (4)
N1—Cu1—O3—C3	−179.09 (14)	O8A^ii^—Cu2—O5—C10	−84.2 (4)
Cu1^i^—Cu1—O3—C3	1.46 (13)	O7—Cu2—O5—C10	92.26 (18)
O2^i^—Cu1—N1—C6	−150.52 (17)	O6^ii^—Cu2—O5—C10	7.0 (4)
O1—Cu1—N1—C6	30.33 (18)	O8^ii^—Cu2—O5—C10	−75.43 (19)
O4^i^—Cu1—N1—C6	−59.76 (18)	O7A—Cu2—O5—C10	82.9 (4)
O3—Cu1—N1—C6	120.91 (17)	N4—Cu2—O5—C10	−174.79 (15)
O2^i^—Cu1—N1—N2	32.91 (13)	Cu2^ii^—Cu2—O5—C10	4.01 (15)
O1—Cu1—N1—N2	−146.24 (12)	C15—N3—N4—C17	−0.2 (2)
O4^i^—Cu1—N1—N2	123.67 (13)	C15—N3—N4—Cu2	−176.20 (12)
O3—Cu1—N1—N2	−55.66 (13)	O8A^ii^—Cu2—N4—C17	41.6 (7)
C6—N1—N2—C8	−0.6 (2)	O7—Cu2—N4—C17	−140.9 (2)
Cu1—N1—N2—C8	176.81 (12)	O6^ii^—Cu2—N4—C17	−50.68 (17)
Cu1^i^—O4—C3—O3	1.4 (3)	O5—Cu2—N4—C17	129.72 (17)
Cu1^i^—O4—C3—C4	−178.07 (13)	O8^ii^—Cu2—N4—C17	39.9 (3)
Cu1—O3—C3—O4	−2.1 (3)	O7A—Cu2—N4—C17	−136.6 (6)
Cu1—O3—C3—C4	177.33 (12)	O8A^ii^—Cu2—N4—N3	−143.5 (7)
N2—N1—C6—C7	0.6 (2)	O7—Cu2—N4—N3	34.0 (2)
Cu1—N1—C6—C7	−176.34 (13)	O6^ii^—Cu2—N4—N3	124.19 (13)
N2—N1—C6—C5	−178.95 (18)	O5—Cu2—N4—N3	−55.42 (14)
Cu1—N1—C6—C5	4.1 (3)	O8^ii^—Cu2—N4—N3	−145.3 (3)
N1—C6—C7—C8	−0.5 (2)	O7A—Cu2—N4—N3	38.3 (6)
C5—C6—C7—C8	179.1 (2)	Cu2^ii^—O6—C10—O5	6.4 (3)
N1—N2—C8—C7	0.3 (2)	Cu2^ii^—O6—C10—C11	−173.03 (13)
N1—N2—C8—C9	179.72 (17)	Cu2—O5—C10—O6	−7.4 (3)
C6—C7—C8—N2	0.1 (2)	Cu2—O5—C10—C11	172.06 (13)
C6—C7—C8—C9	−179.2 (2)	N4—N3—C15—C16	0.3 (2)
O8A^ii^—Cu2—O7A—C12A	8(5)	N4—N3—C15—C14	179.44 (19)
O7—Cu2—O7A—C12A	−154 (6)	N3—C15—C16—C17	−0.3 (2)
O6^ii^—Cu2—O7A—C12A	85.3 (17)	C14—C15—C16—C17	−179.3 (2)
O5—Cu2—O7A—C12A	−82.2 (17)	N3—N4—C17—C16	−0.1 (2)
O8^ii^—Cu2—O7A—C12A	10 (3)	Cu2—N4—C17—C16	175.49 (13)
N4—Cu2—O7A—C12A	180.0 (17)	N3—N4—C17—C18	−178.19 (18)
Cu2^ii^—Cu2—O7A—C12A	0.3 (17)	Cu2—N4—C17—C18	−2.6 (3)
Cu2^ii^—O8A—C12A—O7A	−5(3)	C15—C16—C17—N4	0.3 (2)
Cu2^ii^—O8A—C12A—C13A	175.6 (13)	C15—C16—C17—C18	178.2 (2)

Symmetry codes: (i) −*x*+1, −*y*+1, −*z*+1; (ii) −*x*+1, −*y*, −*z*+1.

### Hydrogen-bond geometry (Å, °)

**Table:**

*D*—H···*A*	*D*—H	H···*A*	*D*···*A*	*D*—H···*A*
N2—H2···O5	0.88	1.93	2.785 (2)	163.
N3—H3···O3	0.88	2	2.847 (2)	163.
